# A Biomimetic Model of Adaptive Contrast Vision Enhancement from Mantis Shrimp

**DOI:** 10.3390/s20164588

**Published:** 2020-08-15

**Authors:** Binbin Zhong, Xin Wang, Xin Gan, Tian Yang, Jun Gao

**Affiliations:** 1 School of Computer and Information, Hefei University of Technology, Hefei 230009, China; 2018110966@mail.hfut.edu.cn (B.Z.); 2018170862@mail.hfut.edu.cn (X.G.); 2017170722@mail.hfut.edu.cn (T.Y.); gaojun@hfut.edu.cn (J.G.); 2 Intelligent Interconnected Systems Laboratory of Anhui Province, Hefei 230009, China

**Keywords:** polarization contrast, eye movements, polarization sensitivity, object detection

## Abstract

Mantis shrimp have complex visual sensors, and thus, they have both color vision and polarization vision, and are adept at using polarization information for visual tasks, such as finding prey. In addition, mantis shrimp, almost unique among animals, can perform three-axis eye movements, such as pitch, yaw, and roll. With this behavior, polarization contrast in their field of view can be adjusted in real time. Inspired by this, we propose a bionic model that can adaptively enhance contrast vision. In this model, a pixel array is used to simulate a compound eye array, and the angle of polarization (AoP) is used as an adjustment mechanism. The polarization information is pre-processed by adjusting the direction of the photosensitive axis point-to-point. Experiments were performed around scenes where the color of the target and the background were similar, or the visibility of the target was low. The influence of the pre-processing model on traditional feature components of polarized light was analyzed. The results show that the model can effectively improve the contrast between the object and the background in the AoP image, enhance the significance of the object, and have important research significance for applications, such as contrast-based object detection.

## 1. Introduction

Due to different visual abilities, the world may present as a thousand looks in the eyes of a thousand creatures. This depends on the biological imaging system and visual information processing mechanism, such as the color vision (CV) that humans rely on, including intensity, wavelength, and saturation [1]. There is a large amount of light with richer dimensional information in the environment [2], which comes from scattered skylight and aquatic space light, surface reflections, or biological polarizers [3,4], i.e., polarized light. Numerous studies have successively shown that many marine creatures, and a few terrestrial creatures, can detect polarized light. They use the polarized light pattern in their field of view as one of the image features for specific visual tasks, such as navigation, intraspecific communication, and contrast enhancement [5], which is called polarization vision (PV). The main polarization components of polarization vision that have been studied are the degree of polarization (DoP) and the angle of polarization (AoP). As there is less circularly polarized light in nature, it will not be considered in the scope of this article. The DoP mentioned refers to the degree of linear polarization (DoLP). The polarization component can be represented by the Stokes vector method [2], as well as intensity (I), another component mentioned in this paper, which is also represented by the Stokes vector.

Polarization information has made contrast vision more effective for living things, which has been confirmed in numerous studies [6]. One of its bases is that common linear polarizers can reduce the amount of perceived scattered light. The scattering of space light in the background is unlike that between the object and the observer, and the difference in polarization information between the two can be useful for object detection [7]. There is a wide range of visual tasks based on polarization contrast. For example, flying insects use polarization contrast to distinguish real water surfaces from virtual water surfaces (such as mirages) [8]. There is also crayfish visual motion detection [9], the predation of transparent zooplankton by marine life [10], and fiddler crab detection of objects in mudflats and other environments [11]. Among those polarization-sensitive creatures, cephalopods and crustaceans are in the majority [12]. Their representatives are octopuses and mantis shrimp, respectively, and there are corresponding bionic studies of these creatures, such as the polarized vision chip made by simulating the octopus retina [13] and the biomedical polarization imaging sensor inspired by the compound eyes of the mantis shrimp [14]. The mantis shrimp, which has outstanding CV and PV (12 color channels [15] and the ability to detect both linearly and circularly polarized light [16]), has attracted our interest.

The mantis shrimp, marine crustaceans of the order Stomatopoda, is extremely aggressive, owing to its sharp, strong, and fast forelimbs and its keen visual acuity. The mantis shrimp has compound eyes, composed of hundreds of visual units (the ommatidia), each of which consists of a cornea covering the lens. There are eight photoreceptor cells (R1–R8) behind the lens, called retinal cells [17]. The eye is divided into three regions: the mid-band region and the dorsal and ventral hemispheres. The microvilli of photoreceptor cells in different regions have different orientations and are generally divided into four categories: 0°, 45°, 90°, and 135°, which indicates that a theoretical four-channel polarization information processing system is available [18]. The electrophysiological evidence of the sensitivity of stomatopods to linearly polarized light [19], as well as the anatomical and neurological characteristics of measurable Stokes parameters [17], further demonstrate the research value of this group in the field of polarization. Recently, bioinspired sensors based on the vision of a mantis shrimp have been developed. The mantis shrimp uses underwater polarization information for navigation purposes [20], which has served as inspiration for several bioinspired underwater navigation sensors and algorithms [21]. Shen et al. adopted the mantis shrimp polarization vision system to realize underwater target detection [22]. Garcia et al. designed a color-polarization imager by functionally mimicking the compound eye of a mantis shrimp [23]. Gruev et al. proposed a polarization sensor with a high dynamic range inspired by the individual photoreceptors of a mantis shrimp [24].

In this article, we focus on the eye movements of mantis shrimp. Mantis shrimp have a variety of eye movements, such as pitching and rolling, and their gaze stabilization is helpful for object detection [25]. Daly et al. studied the movement independence of mantis shrimp eyes [26] and the gaze stabilization under different external actions [27]. Marshall et al. discussed the uniqueness of saccadic eye movements of stomatopod crustacean [28]. However, few specific bionic schemes based on these special eye movements of mantis shrimp have been proposed, especially applications such as contrast vision. There are also studies showing that when they are scanning, the torsion of the eyeball is likely to maximize the acquisition of polarization information in the scene, although how to set the optimal receptor orientation configuration has not yet been solved [29]. Inspired by the special visual behavior of mantis shrimp, we propose a possible mechanism for adaptively pre-processing polarization information. We aim to enhance the polarization contrast between the target and the background to achieve target detection. Our results are directly compared with the polarization components obtained traditionally.

In the subsequent content, Section 2 illustrates the theory of our proposed method. Our measured experimental data are presented in Section 3 to verify our proposed theory. We also analyzed and discussed the experimental results in detail. Finally, in Section 4, we briefly conclude the research work carried out and put forward the prospects for the next step.

## 2. Materials and Methods

### 2.1. Theory of Method

The R1–R8 photoreceptors of the mantis shrimp form the rhabdomeres and the photoreceptors have visual pigment molecules that start the visual process. This type of visual pigment molecule has inherent dichroism, which makes these photoreceptors distinguish and selectively absorb light, i.e., it has polarization sensitivity [30]. When the incoming light is Q, we use the cosine function R(ϕ) to represent the sensitivity of the photoreceptor cell (or receptor) R to polarized light, with

(1) $$R\left(\phi \right)=1+\left[\frac{\left(S_{p}-1\right)}{S_{p}+1}\right]\cos(2\phi -2\phi_{max}),$$

where ϕ is the orientation of the measured polarized light relative to the reference orientation (the e-vector axis), $\phi_{max}$ is the value of ϕ when R(ϕ) has a maximum value, and $S_{p}$ is the effective polarization sensitivity of each receptor ($S_{p}=10$ in the subsequent content of this article),

(2) $$S_{p}=\frac{{\left[R\left(\phi \right)\right]}_{max}}{{\left[R\left(\phi \right)\right]}_{min}}.$$

When the incoming light is partially polarization light with a degree of polarization d, R(ϕ,d) is

(3) $$R\left(\phi ,d\right)=1+\left[\frac{d\left(S_{p}-1\right)}{S_{p}+1}\right]\cos(2\phi -2\phi_{max}).$$

As primarily partially polarized light exists in nature, we only consider Equation (3) [1,31]. Corresponding to the organism, the physical meaning of $\phi_{max}$ is the microvilli orientation of photoreceptor cells. For the four types of receptor orientations of the mantis shrimp, if viewed from the static level, the respective polarization sensitivities are as shown in Equation (4):

(4) $$\{\begin{array}{c} R_{\theta }\left(a,d\right)=1+\left[\frac{d\left(S_{p}-1\right)}{S_{p}+1}\right]\cos(2a-2\phi_{\theta }),\\ \theta \in \left\{0^{{}^{\circ}},45^{{}^{\circ}},90^{{}^{\circ}},135^{{}^{\circ}}\right\}. \end{array}$$

The activity value of receiving polarized light is as shown in Equation (5):

(5) $$S_{\theta }=Q\times R_{\theta }\left(a,d\right),\theta \in \left\{0^{{}^{\circ}},45^{{}^{\circ}},90^{{}^{\circ}},135^{{}^{\circ}}\right\},$$

where $R_{\theta }R_{\theta }$ and $S_{\theta }$ are the sensitivity and activity value of the photoreceptor cells to polarized light, $\phi_{\theta }$ is the orientation of the receptor microvilli, the subscript θ is the deflection angle of the receptor microvilli relative to the horizontal reference orientation, a is the AoP, and Q is the number of original photons (i.e., light intensity).

Theoretically, when the microvilli are oriented parallel to the e-vector axis of the observed polarized light, the extinction coefficient reaches a maximum value, as do the polarization sensitivity and polarized light activity values. Mantis shrimp can achieve four categories of eye motion, including gaze stabilization, scanning, saccade capture, and tracking by pitching, yawing, and twisting their eyes [32]. We conjecture that the process of twisting the eyeball is to find an optimal twist angle to maximize the contrast of the scene in the field of view. Eye movements can turn the directional microvilli of compound eye cells as a whole, and when the microvilli orientation is parallel to the vector orientation of polarized light in the environment, the polarization sensitivity or extinction coefficient will reach a maximum.

**Figure 1:**
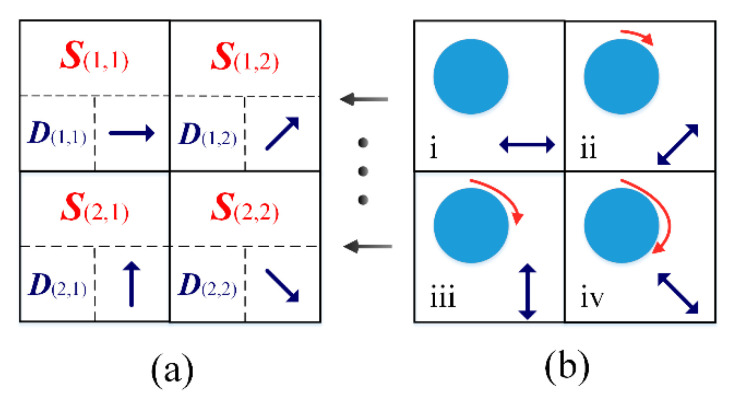
Schematic of 2 × 2 array adaptive mechanism. (**a**) A pixel array of size 2 × 2, where each square has a different polarization state S, D is the degree of polarization (DoP), and the orientation of the arrow is the direction of polarization; (**b**) Our proposed pre-processing array, where there is a rotatable “eyeball” (the blue circle), the principal axis rotation orientation of which corresponds to the angle of polarization (AoP) in (**a**).

Unlike a single scene, where the polarized light is evenly distributed, the polarized light distribution is relatively uneven in the actual environment. The mantis shrimp can only find a comprehensive optimal angle to maximize the polarization information locked in the field of view; however, they cannot be accurate to each point in the spatial position to achieve optimal acquisition [29]. However, inspired by the adaptive PV of the mantis shrimp, we can use a computer to implement adaptive polarization information pre-processing in the form of arrays, when imitating its PV function. In our proposed adaptive mechanism, the core idea is that each point in the visual field image (i.e., each pixel after the sensor is imaged) represents incident light, corresponding to a polarization state, i.e., a specific single polarization direction. The eyeball rotates until the microvilli orientation of its 0° (horizontal reference direction) channel is parallel to the polarization direction at each pixel location. When applying algorithmic processing to an image, all pixels are operated synchronously, thus, this pre-processing array is a pixel-level parallel processing system, as shown in Figure 1.

In this case, $\phi_{0}$ corresponding to each pixel is $\phi_{0}=AoP=\phi_{max}$. We assume that the eyeball is twisted as a whole, thus, the relative orientation of the four-channel microvilli is fixed and Equation (4) can be adjusted to Equation (6):

(6) $$\{\begin{array}{c} R_{\theta }=[1+[\frac{d\left(S_{p}-1\right)}{S_{p}+1}]\cos(2a-2\left(a+\theta \right))],\\ \theta \in \left\{0^{{}^{\circ}},45^{{}^{\circ}},90^{{}^{\circ}},135^{{}^{\circ}}\right\}. \end{array}$$

**Figure 2:**
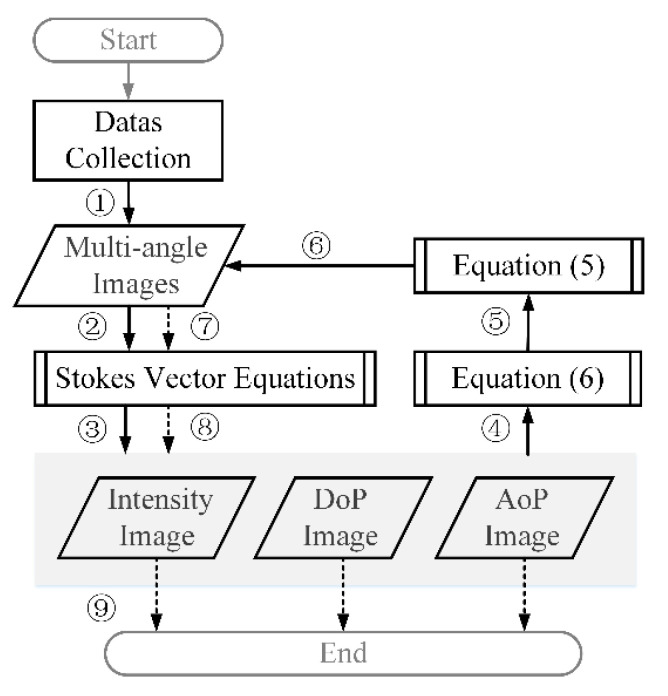
The flowchart of the proposed method.

Furthermore, Equation (5) is updated by using Equation (6). After pre-processing, $I_{\theta }\to S_{\theta },$ $\theta \in \left\{0^{{}^{\circ}},45^{{}^{\circ}},90^{{}^{\circ}},135^{{}^{\circ}}\right\}$. By recalculating the Stokes vector, the polarization component can be obtained. The mantis shrimp does not process light signals by pixel. Here, each pixel is processed independently and treated as a small mantis shrimp sensor, together forming an array. It is time to summarize the concrete implementation of our proposed approach, as shown in Figure 2. The sequence number indicates the sequence of steps for the method, which makes the reconstruction process clearer.

### 2.2. Experimental Schemes of Method

When colors between the object and the background are significantly different, CV has its clear advantages; however, this is outside the scope of this article. To elaborate on the problem, we design experimental scenes that have a similar color or low contrast, such as low visibility. These include three indoor scenes and two outdoor scenes:

i. Vertically oriented linear polarizer and the light emitting diode (LED) panel: In a dark room, we combined a linear polarizer with an LED light source, intended to be a typical polarization object. The light source used a Yongnuo YN300 II LED light. To avoid overexposure when the polarizing imager was imaging, the intensity of the light source was selected as the 01 level. With a 0° orientation of the imager as the reference orientation, the main axis of the linear polarizer was perpendicular to it (i.e., the polarization direction was 90°).
ii. Arbitrarily oriented linear polarizers and the LED panel: This scene was the same as i); however, the major axis orientation of the linear polarizer was arbitrary, i.e., the polarization direction was unknown (simulating a random case).
iii. The plastic socket and the painted wall: In a bright room, the socket and the wall were white, and the socket was covered with a thin and uneven white paint.
iv. The iron box and the green grass: On the outdoor green grass, we placed an iron box with smooth green paint on the surface.
v. The iron box and the land covered by shadows: We made a shadow scene with a visor outdoors and placed the iron box in iv) under the shadow, as a scene with low visibility.

**Figure 3:**
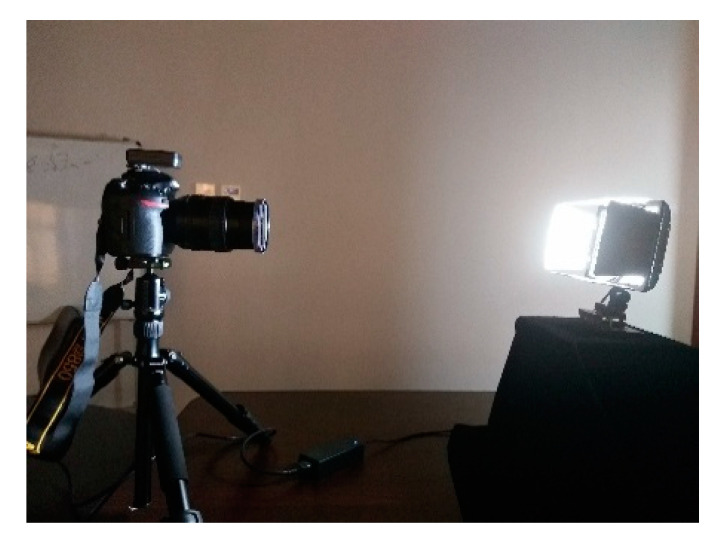
The display of the experimental equipment and environment.

Our experimental site is Hefei University of Technology. Our imaging system consists of a Nikon camera (D850, Nikon Corporation, Tokyo, Japan) with a Nikkor lens (1, Nikon Corporation, Tokyo, Japan) and a rotatable polarizer (Chenter Industries Group Limited, Fuzhou, China), as shown in Figure 3. For each scene, we need to collect the following data: polarized images (with polarizer orientation separately of 0°, 45°, 90°, and 135°) and the original scene images without polarizer.

## 3. Results and Discussion

We collected image data of target scenes under different conditions. At the same time, the experimental results were analyzed and discussed through subjective evaluation and objective quality evaluation.

### 3.1. Contrast Evaluation Indexes

In addition to the subjective evaluation of the experimental results, to more fully analyze the impact of the adaptive mechanism, we also used a variety of contrast evaluation indexes.

#### 3.1.1. Gray Scale Contrast

The difference in image gradation was evaluated as the contrast [33]. The larger the value, the larger the difference between the average gray levels of the two regions being compared, as shown in Equation (7). For convenience of description, it is denoted as GSC,

(7) $$GSC=\frac{\left|{\bar{g}}_{O}-{\bar{g}}_{B}\right|}{{\bar{g}}_{O}+{\bar{g}}_{B}},$$

where ${\bar{g}}_{O}$ and ${\bar{g}}_{B}$ are the average values of the gray levels of the target and background regions, respectively.

#### 3.1.2. Signal-to-Clutter Ratio

The signal-to-clutter ratio treats the background signal as clutter and it is one of the commonly used indicators for object detection. When the target signal is strong and the background clutter is suppressed, the ratio increases, indicating that the method is effective [34]. The ratio is shown in Equation (8), denoted as SCR:

(8) $$SCR=\frac{\left|{\bar{g}}_{O}-{\bar{g}}_{B}\right|}{\sigma_{B}},$$

where $\sigma_{B}$ is the standard deviation of the background area.

#### 3.1.3. Fisher Distance

Fisher distance is often used to evaluate the degree of discrimination between the target and the background, especially for noisy images [35]. Considering the use of the darkroom and shadow in the scene that we tested, the Fisher distance is also used as a contrast index in this study, denoted as FD, as shown in Equation (9):

(9) $$FD=\frac{{\left({\bar{g}}_{O}-{\bar{g}}_{B}\right)}^{2}}{\nu_{O}+\nu_{B}},$$

where $\nu_{O}$ and $\nu_{B}$ are the variances of the target and background regions, respectively.

### 3.2. Experimental Results

**Figure 4:**
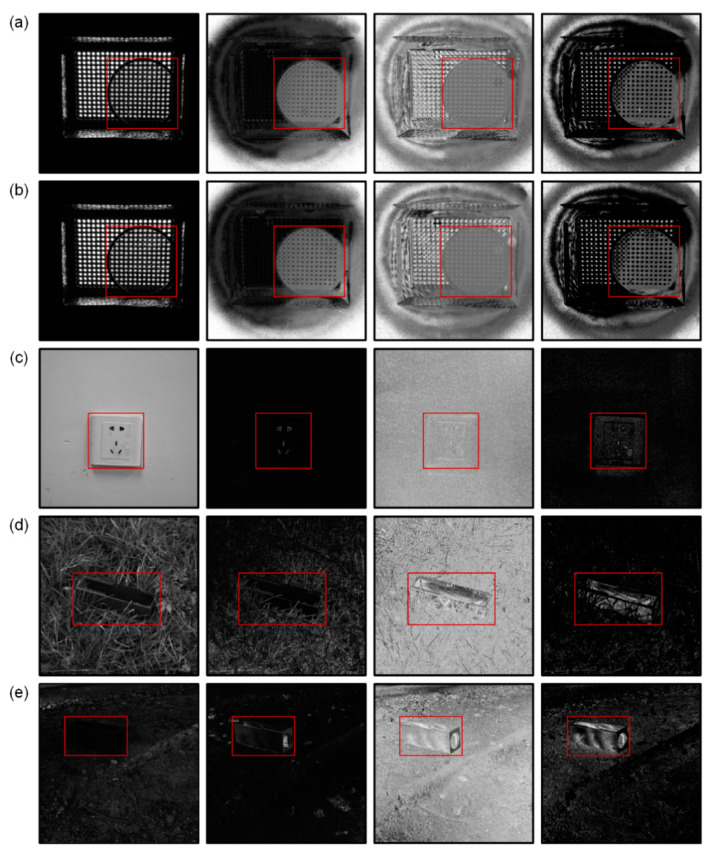
Experimental results (grayscale images) of 5 scenes. Scenes (**a**–**e**) correspond to the scenarios i–v mentioned above. Columns 1–4 (from left to right) are intensity (I), DoP, AoP, and processed AoP, respectively. The red boxes mark the object location of interest in the scene. Scene (**a**) consists of a linear polarizer and a light emitting diode (LED) panel. The linear polarizer is intended to be the object with an AoP of 90°. The configuration of scene (**b**) is similar to (**a**); however, the AoP of the object is unknown. Scene (**c**) consists of the white socket and the wall covered with white paint. Scenes (**d**,**e**) are green iron boxes placed in the green grass and shade, respectively.

It can be seen from Figure 4 that, as shown by previous research results, the original DoP and AoP show advantages in edge contour and texture, respectively. For the first two scenes, as a typical polarization object, the polarization state is almost uniformly distributed and the background is non-polarized, thus, the DoP image is very distinguishable. The polarization states of the latter three application-type scenes were all non-uniformly distributed. The material of the socket in (c) was smooth plastic. Compared with rough walls, the DoP and AoP should have different distinctive features; however, the socket surface was covered with painted materials, thus, the material itself was partially covered. The object in (d) had high concealment because it was obscured by the grass and the target in (e) had low visibility, owing to the shadow coverage. For these cases, the processed AoP image shows stronger object saliency. In addition, some scenes generate artificial polarization information owing to low light intensity. The darker areas in (c), for example, produce higher artificial DoP values. This interference affects the judgment of the target area.

**Figure 5:**
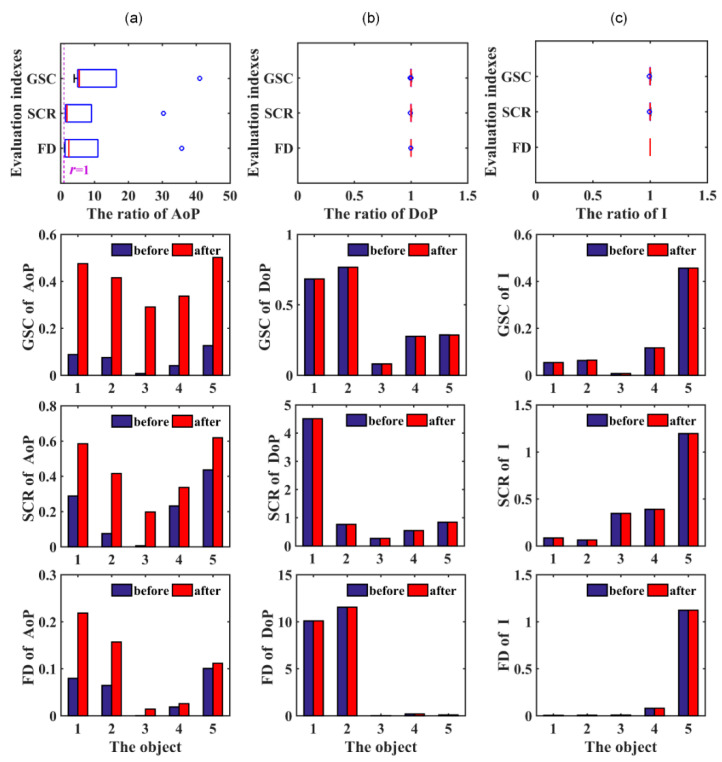
Contrast statistics of the experimental results of 5 scenes. (**a**) shows the results of the contrast value of the AoP in the scene. Row 1 is a box plot. The ordinate represents the different evaluation indexes and the abscissa shows the contrast ratio, before and after the processing method. Rows 2–4 are the contrast results of the AoP image under the gray scale contrast (GSC), the signal-to-clutter ratio (SCR), and the Fisher distance (FD) evaluation indicators. The numbers on the abscissa represent the serial numbers of scenes 1–5. Blue represents the original AoP and red represents the AoP after using the adaptive method. (**b**,**c**) refer to the DoP and I, respectively; the meaning of the graphs are the same as that of (**a**).

Figure 5 shows the experimental results under three evaluation indicators in different forms. Scenes (a), (b), and (c) are experimental data comparisons of AoP, DoP, and I images, before and after using the adaptive method, respectively. Row 1 is a box plot, which shows the overall contrast ratio of the processed image to the original image, i.e., $r=\frac{AoP_{after}}{AoP_{before}}$. If r>1, there is a gain effect. The AoP images of the five scenes satisfy the condition of r>1. Under the GSC index, the maximum multiple is approximately 41.0 and the average multiple is 12.8. For SCR, the maximum and average multiples are approximately 30.3 and 7.4, respectively. For FD, the maximum and average multiples are approximately 35.7 and 8.6, respectively. The DoP and I images do not differ significantly, before and after processing, and the value of r remains at approximately 1. Rows 2–4 of Figure 5 are bar graphs that count the specific contrast values of each component. Blue is the original image and red is the image to which the method in this study is applied. These figures more intuitively show the effect of the adaptive method on the AoP component. The third scene, the socket box on the wall, displays the most obvious optimization effect. The changes of the contrast value of AoP under GSC, SCR, and FD are, respectively, 0.0071→0.2914, 0.0065→0.1973, and 0.0004→0.0143. Even in scene 5 (iron box under the shadow), which had the smallest indicator growth multiples, the contrast of the AoP processed by the adaptive method increased by 37.59%, 41.71%, and 10.51% in turn.

### 3.3. Discussion

After the AoP image of some scenes was adaptively optimized, its edge contours and textures performed slightly worse than other components. The advantage of its contrast feature may be sufficient, depending on the specific visual task. For example, when finding and catching prey, the mantis shrimp may not need to distinguish the stripes of a shell or the complete shape of an octopus. The eye sensor of the mantis shrimp starts eye movements as special signals appearing in the field of vision and enters the alert state. Thus, object saliency or local saliency can be used to quickly find the object, especially for creatures with fast body speeds, such as mantis shrimp. Additionally, the proposed method can improve the contrast between the object and the background in the AoP image, without affecting the DoP and I. This result may have potential value for research on image fusion, based on polarization information.

We consider the cooperation condition of the two eyes of the mantis shrimp. Stomatopod creatures can independently perform eye movements, depending on the visual content they are interested in. Case 1: If two eyes capture the object area and the background area, respectively, they can then obtain the difference in polarization information between the two, almost in real time. However, there are two problems: First, some studies have shown that the spatial positions observed by both eyes when receiving signals should be consistent [18]. Second, many creatures cannot acquire prior knowledge through acquired learning (like humans) to pre-judge the object and the background, but use CV or PV to capture the differences between different regions. It is unclear if the potential “time-share” feature of the mantis shrimp can solve the above problems, as no further research progress is available for reference. Case 2: The eyes of the mantis shrimp will perform consistent eye movements in the same area. They store the captured information in a memory unit and then compare the two pieces of information when scanning to the next area. Although this mechanism is slightly slower than Case 1, the speed of their eyeball scanning or rotation (such as the eye speed of Odontodactylus scyllarus, which can reach 300 °/s [32]) is sufficient to apply for this type of visual task; it also improves the resolution of vision imaging.

## 4. Conclusions

The multiple microvilli orientation of rhabdomeres in the compound eyes of the mantis shrimp enables a multi-channel polarized vision system to receive and process abundant polarized light. There is a potential tendency in their eye movements—the directional microvilli of compound eyes are turned as a whole, as parallel as possible to the observed e-vector axis of polarized light, to obtain maximum polarization sensitivity, thereby improving polarization contrast. Based on this, we referred to the definition of polarization sensitivity and proposed that the AoP direction be pre-set as the receptor orientation. We then combined the characteristics of the computer to process the image pixel by pixel and implemented point-to-point adaptive polarization information pre-processing, in the form of arrays. The pre-processed polarization activity value was used to reshape the polarization component. In scenes with similar colors or high concealment, the method proposed in this study effectively improved the contrast of AoP images and enhanced the saliency of the object; however, it had little effect on the intensity and polarization images. This provides a new perspective to studying the PV mechanism of mantis shrimp and bionic applications, such as object detection. We simultaneously identified that other features (such as the texture) of the processed AoP images do not reach our expectations. The integration of the advantages of various components into one is still a subject to be solved. Our future work is expected to include image fusion, based on polarization information.
